# Accreditation in India: Pathways and Mechanisms

**DOI:** 10.1080/21614083.2018.1454251

**Published:** 2018-04-04

**Authors:** Swaptagni Das, Manan Shah, Amey Mane, Vishal Goyal, Vikram Singh, Jayesh Lele

**Affiliations:** a Janssen Medical Affairs, Johnson & Johnson Pvt Ltd, Mumbai, India; b Secretary, Indian Medical Association – National Hospital Board of India, Mumbai, India

**Keywords:** Accreditation, continuing medical education (CME), credits, medical councils, re-licensure, re-registration

## Abstract

Continuing medical education (CME) is a valuable mechanism to update physicians’ knowledge with ever-increasing plethora of contemporary advances within medical fraternity. Over time, scope of CME has seen change from simple clinical updates to comprehensive continuing professional development (CPD), which is accomplished with help of accredited CME programmes. The Medical Council of India, in 2011, made a mandatory resolution for doctors to attend minimum of 30 hours of CME/5 years to ensure recertification. Authorised accreditation councils and licensing authorities award CME credits for maintenance of physicians’ licensures. To date, in India, only 9 of 26 State Medical Councils have made re-registration mandatory. Although CME events benefit healthcare professionals by improving their proficiency and awareness, costs even to attend such interventions may be prohibitive. Despite financial help being received through grants and sponsorships, ethics of industry-sponsored CME remains a matter of debate. However, over past 10 years, pharmaceutical companies have started going beyond basic product information in order to focus on building physicians’ knowledge in various therapeutic areas. Though CME credit system and criteria for re-licensure for medical practice in India are evolving at a rapid pace, there is a need for harmonisation and robust implementation across all states in India.

## Introduction

There is an unceasing requirement for advances in clinical practices and patient care, thereby changing the concepts and approaches towards diagnosis, prevention and treatment. It therefore becomes a primary responsibility for physicians to keep abreast of the ever-increasing advances in the medical field, requiring constant updating of medical knowledge to provide the highest quality of care to their patients [1–3]. CME is an educational activity that contributes to maintaining, improving and updating a physician’s knowledge, expertise and professional performance.

With the passage of time, the scope of CME has seen a change from simple clinical updates to CPD which additionally brings about behavioural changes, societal and managerial skills, imparts the value of ethics and assists in achieving the primary goal of providing optimal patient care [4]. Thus, CPD is not only a commitment to enhance structured skills or development of personal or professional competence, but it also acknowledges the multidisciplinary conditions of patient care [3]. Accredited CME plays an important role in contributing to the professional development of healthcare professionals (HCPs) [2]. The process of accreditation involves review and evaluation of an educational programme by a designated authority, using a set of clearly defined criteria and procedures [5]. Accreditation of CME is the recognition and certification of CME organised by an institution or organisation that meets defined standards, which in turn encourages institutional development. Live events have become the preferred mode of CME for most professional organisations.

## Data collection

Data were mainly collected from a search of review articles and health reports over the last five years; most recent data were prioritised. Table and charts were developed from the review articles and/or health reports. The authors also attended discussion sessions with the councils authorised to regulate CME and information thus elicited is also presented here.

### CME programme in India

The Medical Council of India (MCI) is the national governing body responsible for the establishment and maintenance of high-standard medical education as well as recognition of medical qualifications. The MCI and the 26 State Medical Councils (SMCs) are the major CME regulators in India. They also accredit the CME events and allocate credits to the HCPs. Other CME regulators include the Indian Medical Association (IMA), Association of Physicians of India and individual certification programmes. Furthermore, the MCI has advised medical schools to develop an interest in CME [6].

In India, up to March 2016, of the total population of 1,341,607,617 [7], the MCI lists 978,735 as registered medical practitioners [8]. As of June 2017, India has 460 undergraduate medical schools with an annual intake capacity of 64,725 candidates [9]. Medical schools with competency-based curricula can help develop clinical and problem-solving skills in trained health professionals, thus updating their knowledge on advances in this field [6]. In 2002, with approval from the Government of India, the MCI issued a gazette notification (Code of Medical Ethics regulations [Amended up to 8 October 2016]), related to professional conduct, etiquette and ethics for registered medical practitioners. Regulation 1.2.3 states:

A physician should participate in professional meetings as part of a CME programme, for at least 30 hours every five years, organised by reputed professional academic bodies or any other authorised organisations. The compliance of this requirement shall be informed regularly to MCI or SMCs as the case may be.

In April 2011, the MCI passed a resolution on CME, by which it was made mandatory for all doctors to attend a minimum of 30 hours of CME in every 5 years, failing which their registration to practice would be suspended [10].

Most medical associations in the western world have also made CME participation obligatory for registered medical practitioners in order to maintain their medical licensure. Over the past few years, Singapore [11], Australia [12], South Africa [13], Pakistan [14] and Bhutan [15] have mandated CPD/CME, while Bangladesh [16], Malaysia [17] and others are either in the process of, or about to initiate, enforcement of the credit hour system (Table 1). A survey conducted among 751 physicians from 8 medical specialties across India demonstrated that most of the participating physicians in CME programmes were men (86%), of whom 40% were between 41 and 50 years of age. Most of the physicians (72%) had more than 10 years of clinical experience [18].

**Table 1.** CME accreditation criteria – other countries.

Country	Governing body	Guideline	No. of credits/frequency	Participation	Penalty	Drawbacks
Malaysia [17]	Malaysian Medical Council/Malaysian Medical Association	Malaysian Medical Council CME Grading system	Per Malaysian Medical Council CME Grading system	Voluntary but strongly encouraged	None	No robust accreditation system
China [16]	National CME Commission/Provincial CME commission/Municipal CME Commission	-	25 credit hours/year (Category 1: 5–10 credit hours [1 credit = 2–3 learning courses]; Category 2: 15–20 credit hours [self-learning, project and/or hospital-based learning activities])	Mandatory	-	Evaluation is less frequent.
Nepal [16]	-	-	100 electronic continuing medical credits/3 years.	Mandatory	-	-
Bhutan [15]	-	Bhutan Medical and Health Council Regulations 2005 (Section 16.2)	30 credits/5 years (GPs: 6 credits/year; specialists: 3 credits/year; non-practitioners: 15 credits/5 years) (3 working hours = 1 credit)	Mandatory	-	-
Bangladesh [16]	-	-	Not available	Not mandatory	-	No systematic approach
Pakistan [14]	-	-	GPs: 5 credit hours/year; Specialists: 10 credit hours/year	Mandatory	-	-
South Africa [13]	Health Professional Council of South Africa (HPCSA)	HPCSA guidelines	60 credits/2 years (of which 10 should be on ethics, human rights and medical law)	Mandatory	Membership suspended after 6 months of non-compliance. Restoration granted after submitting proof of accruing mandatory credits.	-
Brazil [16]	Respective or medical associations	-	Determined by individual specialty association	Mandatory	-	-
Russia [16]	Medical education institutions	-	Every 5 years in respective specialty	Mandatory	-	-
Singapore [11]	Singapore Medical Council	-	25 CME points/year (20% of which are specific to faculty) (1 CME credit = 1 h of CME activity)	Mandatory	Removal of licence	-
Canada [16]	Federation of Medical Regulatory Authorities of Canada	Maintenance of Certification (MOC) Program; Maintenance of Proficiency+ (Mainpro+)	400 credits/5 years (minimum of 40 credits/year)	Mandatory	Possible removal of licence	-
Australia [12]	Medical Board of Australia	-	50 credit hours/year	Mandatory	Suspension from register	-

CME, continuing medical education; GP, general practitioner; HPCSA, Health Professional Council of South Africa.

With growing numbers of registered doctors, in addition to providing updates, CME also helps in ensuring their recertification [6]. Authorised accreditation councils and licensing authorities award CME credits for maintenance of licensure of registered medical practitioners. As of 2016, nine SMCs have made re-registration for medical licensure mandatory for medical practitioners (Table 2); other state councils are expected to enact similar measures in the coming years [19]. However, non-accredited CME events, which are generally provided by pharmaceutical companies, compete with CME accredited events.

**Table 2.** State Medical Councils that made re-registration of medical licence mandatory until 2016.

Sr. No.	State Medical Council	Credit hours system
1	Goa Medical Council [22]	30 credit hours every 5 years
2	Karnataka Medical Council [23]	
3	Kerala Medical Council [24]	
4	Maharashtra Medical Council [25]	
5	Rajasthan Medical Council [26]	
6	Tamil Nadu Medical Council [27]	
7	Uttar Pradesh Medical Council [28]	
8	Gujarat Medical Council [29]	30 credit hours/year up to 150 credit hours every 5 years
9	Punjab Medical Council [30]	50 credit hours every 5 years (10/year)

**Figure 1.**
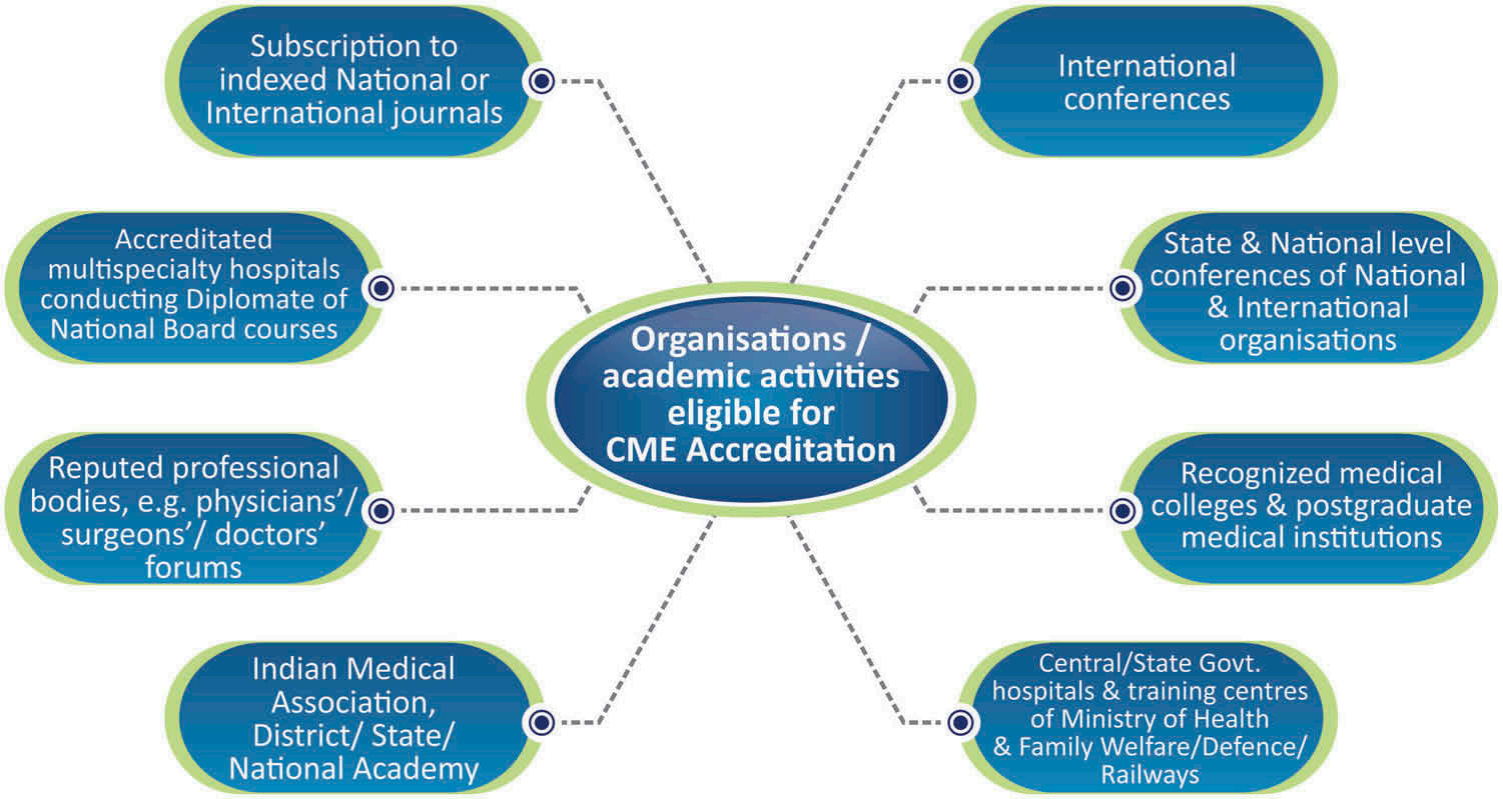
Organisations/academic activities eligible for CME accreditation. CME, Continuing medical education.

To date, the Maharashtra Medical Council (MMC) has accredited 5471 speakers [20] and 332 organisations [21], which arrange almost 25 live events each week throughout Maharashtra (Figure 1). Accredited speakers are assigned a unique speaker code for ease of access to CME organisers.

### Criteria for CME accreditation by SMCs in India [22–30]

**Figure 2.**
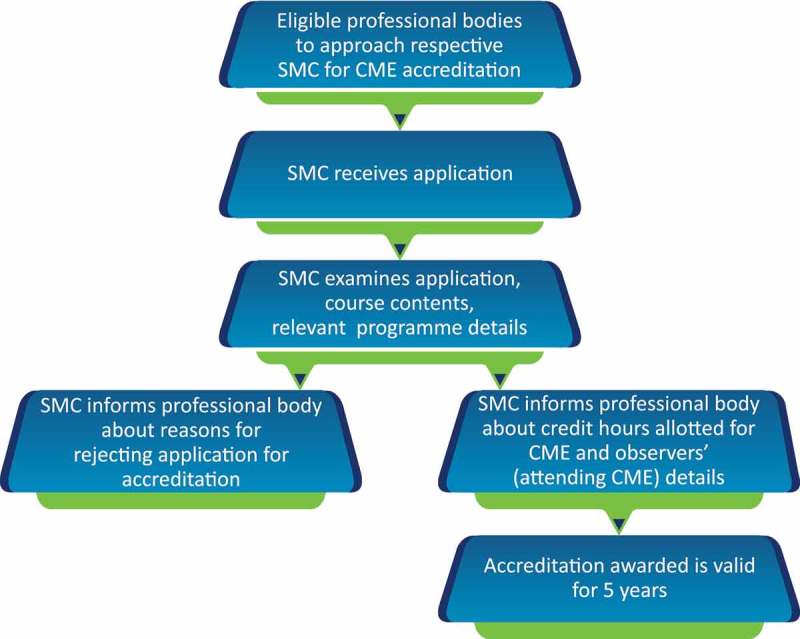
Procedure of CME accreditation. CME, Continuing medical education; SMC, State Medical Council.

- The SMC solely takes the decision to award credit hours which depends on the quality of the subject matter, and the status and credibility of the speaker.
- The office bearers of the organisation planning to provide the CME are expected to apply for accreditation to their respective SMC.
- Besides the topic and duration of presentation, the complete schedule and transcripts of the event, including faculty names, designations, qualifications and their country of residence, are to accompany the application (Figure 2).
- Different SMCs in India showed highly varying criteria for CME credit hours; e.g. Chhattisgarh Medical Council [31], Madhya Pradesh Medical Council [32] and Himachal Pradesh Medical Council [33] award two credit hours for a half-day event as compared with one credit hour by the Tamil Nadu Medical Council [27]. Different SMCs award variable credit points for conducting a CME programme/workshop/conference, publication of medical textbooks or papers, and for attending/presenting at conferences. Other criteria for awarding CME credit points include subscription to journals, postgraduate courses for doctors, guest lectures, attendance at a guest lecture by foreign faculty, and online module certification. (Figure 3(a)–3(e)).
- Accredited professional bodies, such as the IMA, which arrange CME programmes regularly, have to inform the SMC of the date and time of the event in advance, so that the SMC can assign an observer.
- Upon validation of the organisation and event credentials, the SMC issues a certificate of accreditation.
- The providing organisation has to make prior arrangements for publicity to reach out to the target group of participants/delegates.
- The providing organisation must not award certificates to the attending participants/delegates until the last day, i.e. after completion of the CME programme.
- The providing organisation has to ensure that the list of delegates and their feedback are sent to the accrediting SMC. A distinct list of delegates belonging to the various SMCs should be submitted to the accrediting SMC.
- The accreditation certificate of the issuing organisation will be revoked if found to have been fabricated.

* On completion of the CME programme, a brief summary/report (both hard copy as well as soft copy) along with the list of delegates and their registration numbers is to be sent to the SMC.

**Figure 3a.**
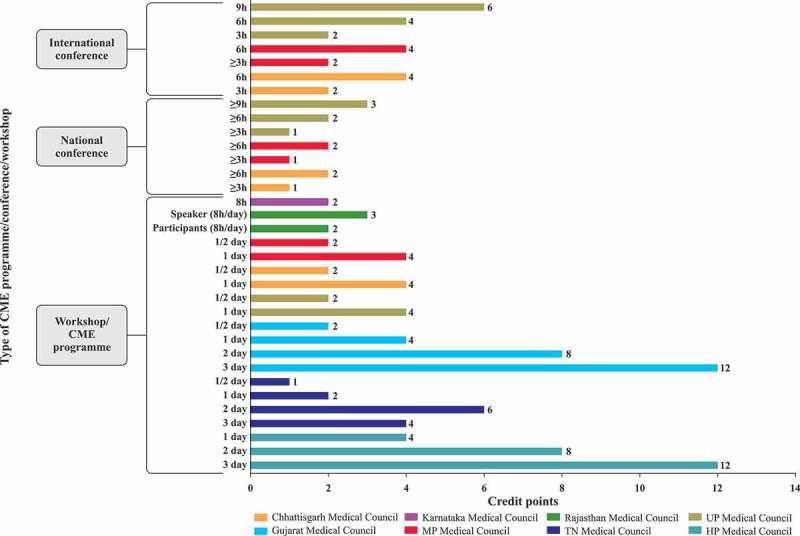
Credit points awarded for CME programme/workshop/conference [23,26–29,31,32]. CME, continuing medical education; h, hours; HP, Himachal Pradesh; MP, Madhya Pradesh; TN, Tamil Nadu; UP, Uttar Pradesh

**Figure 3b.**
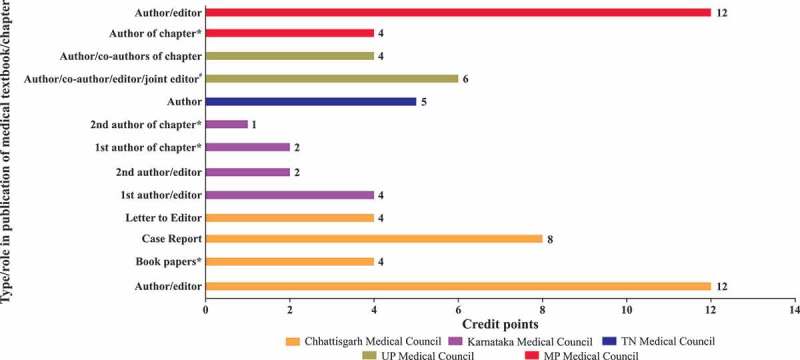
Credit points awarded for publication of medical textbooks [23,27,28,31,32]. * Published in International Indexed Journals; # Published by professional bodies.

**Figure 3c.**
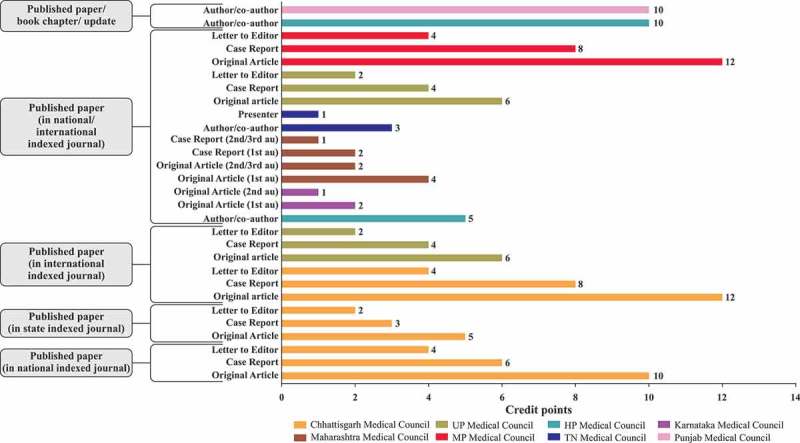
Credit points awarded for published papers [23,25,27,28,30–33]. au, author; UP, Uttar Pradesh; HP, Himachal Pradesh; MP, Madhya Pradesh; TN, Tamil Nadu

**Figure 3d.**
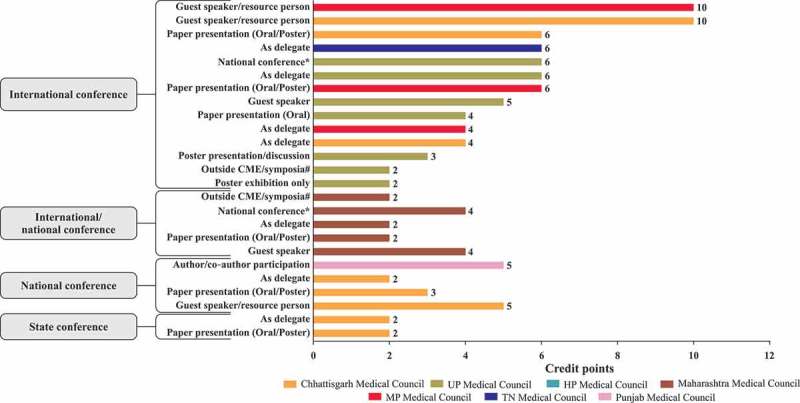
Credit points awarded for attending/presenting in conferences [25,27,28,30–33]. UP, Uttar Pradesh; HP, Himachal Pradesh; MP, Madhya Pradesh; TN, Tamil Nadu; # organised by recognized professional bodies; * organised by National Professional Association.

**Figure 3e.**
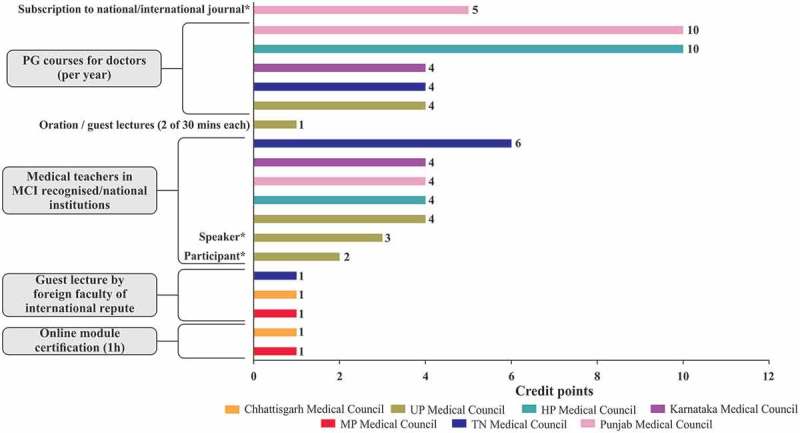
Other major criteria for awarding CME credit points [23,27,28,30–33]. CME, continuing medical education; HP, Himachal Pradesh; MCI, Medical Council of India; MP, Madhya Pradesh; TN, Tamil Nadu; UP, Uttar Pradesh * conference/CME programme/workshop ≥8h/day. Note: Kerala Medical Council, CME accreditation shall be at the rate of 40% of actual CME hours on the first day and 30% for subsequent days for CME exceeding 2 h duration and 25% for CME of less than 2 hours duration subject to maximum of 3 accredited hours per day; minimum duration of CME programme should not be less than 1 h; maximum of 3 h only can be accumulated in a year from programme of less than 2 hours duration [24].

### Continued medical education activities not accredited by SMCs

The CME events organised by the following are not accredited by SMCs [23–31]:

- Drug/equipment company for promotion of their product
- Individual nursing homes/hospitals for marketing purposes
- Those for self promotion or advertisement
- Non-registered professional bodies
- Credit hours are not given for live operative workshops conducted by foreign delegates unless they obtain provisional registration from MCI.

Through the agency of the World Health Organization, CME programmes have been held in many cities in India (*South*: Belgaum, Mangalore, Madurai, Manipal and Tirunelveli; *North*: Dharamshala, Aligarh and Muzaffarnagar, Bhopal; *West*: Nagpur and Jaipur; *East*: Cuttack, Patna and Guwahati) [34].

## Discussion

It is imperative that the medical fraternity keeps pace with evolving science in relation to patient care. CME is a valuable mechanism to update available knowledge with ever-increasing contemporary advances in drug delivery systems, treatment modalities, patient management skills, therapy modelling and diagnosis [6]. Educational CME topics are based on areas like disease overview, disease progression, disease management, recent updates and/ or new concepts shared in International and National conferences and managing the complications of diseases, among other topics. India, with support from its CME system, aims for state-of-the-art quality care for its patients. CME can prove to be the backbone of quality assurance in hospitals and thus, can effectively improve care by refining physicians' performance. New models of CME not only impart knowledge, but also transform physicians’ perspective, and may even play a role in organisational improvement [35]. In order to keep up with the interntional *standards*, it is essential that the various stakeholders strive to work in co-ordination and unison to improvise and implementthis system. It is imperative to guide CME providers to aim at assisting physicians to achieve CPD while assuring enhanced patient outcomes. Currently, the system functions via state and regional legislative bodies that accreditate CME programmes and award credit hours to registered medical practitioners to encourage them to keep up to date with the latest advances. However, the system lacks harmonisation due to the variation in criteria put forth by individual SMCs. There is a need for synchronisation with international CME standards [19]. Forming policies and implementing CME programmes is a daunting task. Obstacles include, but are not limited to, variation in CME accreditation standards across states, disparity among mandatory CME credit regulations, and uncertainty regarding recognition of online CME, as well as funding policies [6].

Despite implementation of several CME programmes in India, doctors in many rural areas have little or no access to these courses. A lack of mandatory or other incentives also limits CME opportunities in the developing world. Although there is an established code to complete 30 hours of CME/5 years, only about 20% of India’s doctors comply with this, as it is not legally binding [34].

Engaging in CPD has become a fundamental process for revalidation and re-licensure for practising physicians [3]. The MCI has directed and is ensuring that medical schools undertake the responsibility for robust implementation of CME as a supplementary function [6]. Although the regulatory mandate stipulates that a deficit in accomplishing the necessary credit requirements can result in termination of registration for practice (licensure), no enforcement has yet been established, although nine SMCs charge an insignificant monetary penalty for delay in compliance [19].

In India, didactic, live lectures are the prime and generally accepted mode of accredited CME. Although in a preliminary phase, the concept of online learning (web-based CME) is emerging gradually, with some SMCs conferring credits for accreditation. However, a major constraint encountered by most CME providers in India is the scarcity of experts in the relevant medical fields. In order to ensure that CME results in higher impact and acceptance, there is a need for more formally trained professional teachers. The speakers are expected to have no conflicting interests and should be specialists in their field, but this is often not achieved as they lack the expertise to engage the learners effectively due to inadequate soft skills [19].

Certain SMCs in India have accepted and implemented this system successfully. However, the mandatory CME credit obligation imposes an added burden on the medical fraternity to organise CME activities. Even though CME events benefit HCPs by improving their efficiency, the costs involved in attending educational sessions are high. This has been a major hindrance and parent organisations and the pharmaceutical industry may be approached for funding [36].

Most CME is sponsored by the pharma/drug companies. Financial help in organising CME is also received through grants and sponsorships from organisations such as MCI, international organisations (e.g. UNICEF) and private firms such as Indian generic drugs companies, as well as the Indian Ministry of Health [37]. However, the ethics of industry-sponsored CME remains contentious [6,38]. Thus, depending on the prevalent national regulations, the scope and sources of funding may vary [39]. In the US, nearly 60% of the funds required for conducting CME are obtained from pharmaceutical companies [40]. In India, MCI regulations (2002) discourage pharmaceutical and device companies from providing programmes which directly promote their products in CME content [19]. However, the pharmaceutical industry has, in its own way, contributed towards the progress and expansion of the CME system. In the international scenario, medical education companies have a greater level of acceptance, mainly because of their ability to provide trained experts for educational events. The responsibility of these experts is to ensure that CME is interactive and motivating. They are able to deliver comprehensive CME programmes that integrate expertise from various sources including faculty from diverse organisations, presentations from different specialists and different conferences [6].

Over the last decade, the outlook of pharmaceutical companies towards CME has evolved from being “product-orientated” to focusing on physicians’ knowledge in the relevant therapeutic area. The content is more “learner-based” than “activity-based” and has seen a shift from the use of product names to generic names. Pharmaceutical companies seek to recognise physicians’ gaps in knowledge, competence and performance and aim to update physicians to the most recent research developments in their therapeutic areas.The content they develop from CMEs is evidence-based, scientifically rigorous and therapeutically balanced. This change in outlook ascertains updating physicians to the most recent research developments in their therapeutic areas, which in turn results in improved patient care.

Without industrial funding, organisers claim that delivering CME programmes just from registration fees is practically impossible. Most medical practitioners find the fees exorbitant, especially when travel expenses are involved. Some studies reveal that financial support received from the industry results in higher participation from medical practitioners [41]. Tabas et al. [42] reported that 62% of participants were in agreement that commercial funding was essential to reduce the overall expenses incurred in participating in and attending CME activities. Similar findings were observed from the US [41] and Scotland [43].

The MCI has added an amendment, “Indian Medical Council (Professional Conduct, Etiquette and Ethics) (Amendment) Regulations, 2009 – Part-I”, to its Indian Medical Council (Professional Conduct, Etiquette and Ethics) Regulations, 2002, with respect to unethical acts. The newly added clause 6.8 in chapter 6 discusses the “Code of conduct for doctors and professional association of doctors in their relationship with pharmaceutical and allied health sector industry”. The medical practitioner is discouraged from accepting from any pharmaceutical or allied healthcare industry source any travel facility inside or outside the country (e.g. paid vacations or attending conferences) for self and family members (sub-clause 6.8.1). Also, the clause discourages a medical practitioner from receiving either hospitality or gifts from pharmaceutical companies. A medical practitioner is expected to obey and abide by the stipulations for his/her actions to be considered ethical [44].

## Conclusion

CME plays a vital role in providing updates in scientific research in addition to the new advances in clinical skills necessary for improving patient care. With a population of 1.3 billion, India has nearly 980,000 registered medical practitioners and 460 medical schools with an annual student intake capacity of 64,725 candidates. The medical education licensing authorities emphasise the importance of the requirement for CME credit hours for ongoing registration of clinical practitioners. The CME credit system and criteria for re-licensure for medical practice are evolving rapidly. However, a robust implementation of the system with harmonisation across all states is essential. A collaborative effort by the industry, professional associations, regulators and accreditors may be instrumental in improving the processes and outcomes of medical education.
